# FK506 Induces Ligand-Independent Activation of the Bone Morphogenetic Protein Pathway and Osteogenesis

**DOI:** 10.3390/ijms20081900

**Published:** 2019-04-17

**Authors:** Sreedhara Sangadala, Emily J. Devereaux, Steven M. Presciutti, Scott D. Boden, Nick J. Willet

**Affiliations:** 1 Atlanta VA Medical Center, Decatur, GA 30033, USA; steven.presciutti@emory.edu; 2 Department of Orthopaedics, Emory University School of Medicine, Atlanta, GA 30322, USA; emily.foerster@emory.edu (E.J.D.); sboden@emory.edu (S.D.B.)

**Keywords:** BMP-2, tacrolimus, osteogenesis, small molecule

## Abstract

Osteoinductive bone morphogenetic proteins (BMPs), including BMP-2, have a unique capability of mediating bone formation both in orthotopic and ectopic locations. Immunosuppresive macrolides have been shown to potentiate BMP-2 activity through FKBP12, but these have yet to translate to effective osteoinductive therapies. Herein, we show the osteogenic activity of FK506 as a stand-alone agent in direct comparison to BMP-2 both in vitro and in vivo. FK506 was capable of producing stand-alone alkaline phosphatase induction in C2C12 cells comparable to that seen with rhBMP-2. FK506 treatment activated the BMP receptor, as shown by increased pSmad1/5 levels, and produced significantly higher mRNA levels of the early response genes in BMP and TGF-β pathways. Additionally, the FK506 induction of alkaline phosphatase was shown to be resistant to Noggin treatment. In vivo osteogenic activity of FK506 was tested by local delivery on a collagen sponge in an ectopic subcutaneous implantation model in the rat. Dose responses of FK506 showed increasing levels of ectopic mineralization comparable to the mineral volume produced by BMP-2 delivery. These findings suggest that the use of FK506 can enhance osteoblastic differentiation in vitro and can induce mineralization when delivered locally in vivo.

## 1. Introduction

BMPs (Bone Morphogenetic Proteins) are the key proteins mediating mesenchymal stromal cell (MSC) recruitment, differentiation, and maturation into osteoblasts [1,2]. Since the Food and Drug Administration (FDA) approval of recombinant human (rh) BMP-2 in 2002, it has been widely adopted clinically. rhBMP-2 delivery demonstrates consistent bone formation, but has also been associated with several local adverse side effects including inflammation, vascular permeability, seromas, hematomas, and nerve root irritation [3,4,5,6,7,8]. Most of the initial BMP-2 dose is released from its collagen carrier in the first several days, leaving only a small amount of residual BMP-2 to initiate differentiation of osteoprogenitors as they arrive during the first two weeks. Simply lowering the initial BMP dose does not solve the clinical problems, as the residual dose would also drop below the threshold of the necessary dose for consistent bone induction in humans.

**Figure 1:**
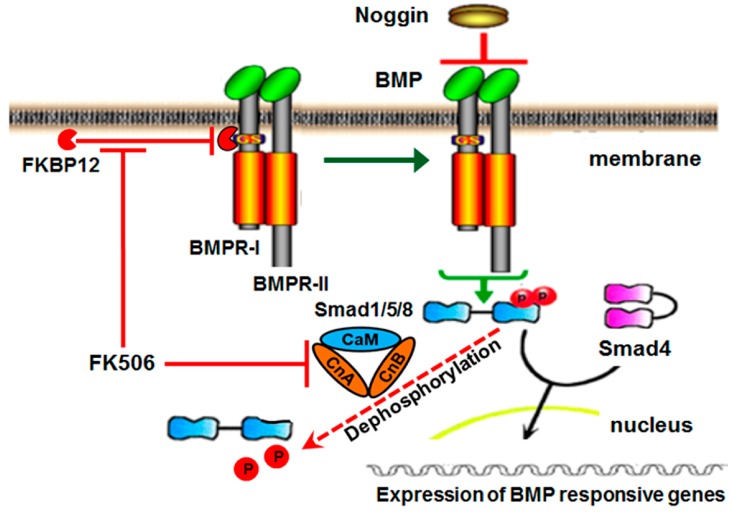
Bone morphogenetic protein (BMP) signaling pathway and inhibition by Noggin. The BMP dimer binds to BMP receptor type II, which recruits type I receptors so that a hetero-tetramer is formed with two receptors of each type. The proximity of the receptors allows the type II receptor to phosphorylate the type I receptor. Activated type I receptors phosphorylate R-smads or receptor-regulated Smads (Smad1/5/8), which form complexes with Smad4. Activated Smad complexes regulate gene expression of several target genes. Calcineurin is a Ca++–calmodulin-dependent serine/threonine protein phosphatase. It is a heterodimer of a ~60 kDa catalytic subunit, calcineurin A (CnA), and a 19 kDa regulatory subunit, calcineurin B (CnB), and has been implicated in the BMP pathway as depicted.

The current first-generation clinical strategy for BMP-induced bone formation involves the traditional ligand-dependent activation of the BMP/Smad pathway, which begins when rhBMP protein binds to the receptor complex. BMP receptors (BMPRs) are the transmembrane serine threonine kinase receptors, classified as type I (BMPRI) or type II (BMPRII) based on their sequence homology [9]. BMPRI, when activated by BMPRII upon ligand binding, phosphorylates receptor-associated Smad proteins. The activated pSmads associate with the common Smad, Smad4, and proceed to the nucleus to turn on the target gene transcription (Figure 1) [10,11]. Phosphorylated Smad1/5 can be dephosphorylated by calcineurin [12,13].

Recently, an intracellular BMP repressor that targets BMPRI has been identified [14]. The immunophilin FK-binding-protein-12 (FKBP12) is an intracellular BMP inhibitor and serves in preventing leaky signaling under sub-optimal ligand concentrations. FKBP12 prevents the type II receptor from phosphorylating the type I receptor, and thus prevents the association and subsequent phosphorylation of cytosolic receptor Smads [14]. A type I receptor mutation in Fibrodysplasia Ossificans Progressiva (FOP) reduces FKBP12 binding to receptor and results in leaky BMP signaling in the absence of ligand, leading to spontaneous ossification, indicative of a potential therapeutic route for osteogenesis through targeting FKBP12 [15].

FK506 is a lipophilic 23-member macrolide lactone isolated from *Streptomyces tsukubaensis* (molecular weight of 803.5 Da). It is an immunosuppressant that is widely used in transplant patients and is typically delivered systemically (with a half-life of 8.7–11.3 h) [16,17]. FK506 induces its effects in part by binding to FKBP12, inhibiting calcineurin activity, and preventing T cell proliferation [18]. Most members of the FKBP family bind to FK506 and show peptidylprolyl cis/trans isomerase (PPIase) activity. Small-sized FKBP family members contain the FK506-binding domain, while the large FKBPs also possess extra domains, such as tetratricopeptide repeat domains, and calmodulin binding and transmembrane motifs [18].

In addition to the immunosuppressive activity, FK506 has been shown to exercise a variety of actions on bone metabolism and osteogenesis. In vitro, FK506 can enhance BMP-2 activity and promote osteogenic differentiation of various cell lines including rat MSCs, mouse pre-osteoblasts, and limb bud cells, among others [19,20,21,22,23]. In vivo, when administered systemically in a continuous manner, FK506 causes osteopenia in mice, rats, and humans [14,24]. However, both bone healing and induction of ectopic mineralization have been shown to be enhanced by systemic delivery of FK506 [25,26,27,28]. Further, when administered locally in combination with BMPs (or *BMP* gene therapy), FK506 enhances BMP-induced mineralization [29]. These data present compelling utility for FK506 as an osteoinductive stimulus, but the local delivery of FK506 in a stand-alone manner has not been performed.

Our objective was to test the osteogenic activity of FK506 as a stand-alone agent both in vitro and in vivo. We hypothesized that the transient activation of BMP signaling through FK506 delivery would initiate the local osteoinductive cascade for induction of bone without adjunctive recombinant BMP or implanted MSCs. Herein, we performed in vitro dose–response studies with FK506 to induce an osteogenic response in comparison to the dose response of BMP-2 in vitro, and tested the local delivery of escalating doses of FK506 delivered locally on a collagen sponge in an ectopic mineralization assay in vivo.

## 2. Results

### 2.1. Macrolide Enhancement of BMP Signaling in C2C12 Cells

**Figure 2:**
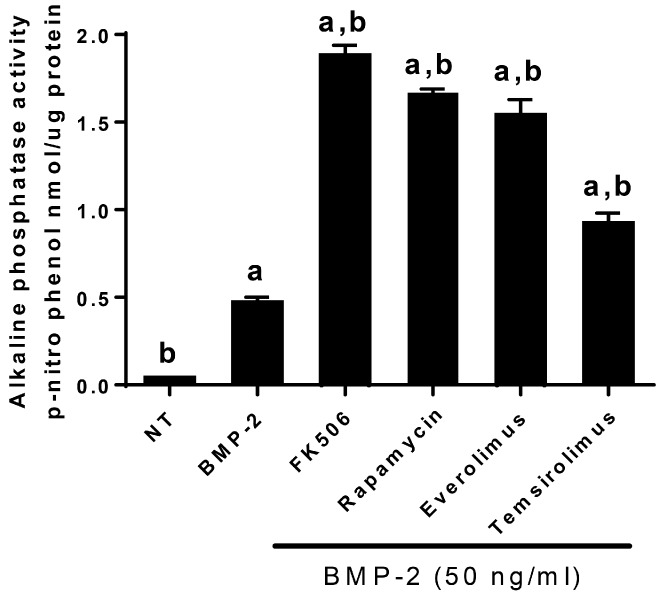
Enhancement of BMP-2-induced ALP enzyme activity by a family of macrolides (FK506, rapamycin, everolimus, and temsirolimus) that were computationally predicted to interact with BMP receptor type I (BMPRI), displace FKBP12, and potentially enhance BMP signaling. FK506 exhibited the strongest ability of the macrolide family to enhance BMP-2-induced alkaline phosphatase activity after 72 h of treatment. Data points of luciferase activities were determined in triplicate (*n* = 3). The labelings of a (BMP-2 alone control) and b (no treatment control) denote statistical significance (*p* < 0.05), determined as described in “Materials and Methods,” between the indicated treatments.

We used an in vitro screening assay in pluripotent C2C12 cells to measure the abilities of various macrolides (FK506, rapamycin, everolimus, and temsirolimus) (at 5 µM concentration) to enhance BMP-2 induction of alkaline phosphatase as an early marker of osteogenic differentiation. All experiments were performed with a sample size *n* = 3. All the compounds are structurally related to the FK506 macrolide lactone. FK506 exhibited the strongest ability of the macrolide family to enhance BMP-2-induced alkaline phosphatase activity after 72 h of treatment, and was significantly (*p* < 0.05) greater than both BMP-2 alone and no-treatment (NT) controls (Figure 2). All the macrolides exhibited 2–4 fold enhancement of sub-optimal BMP-2-induced alkaline phosphatase activity, and were significantly (*p* < 0.05) greater than the BMP-2 alone and NT controls. The control samples were treated with the solvent, DMSO, at lower than 0.01% in culture media. DMSO alone has no effect on either cell morphology or differentiation at the concentrations tested in these experiments.

### 2.2. FK506 Independently Mimics BMP-2 Induction of Alkaline Phosphatase Activity

**Figure 3:**
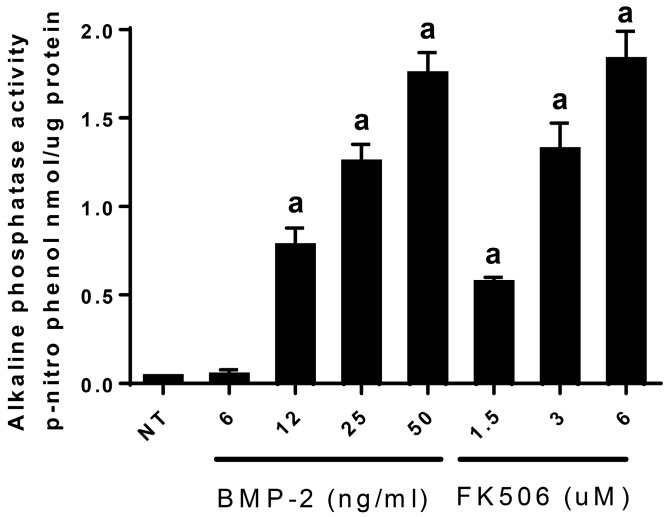
FK506 independently mimics BMP-2 induction of alkaline phosphatase activity. FK506 (6 µM) was capable of producing a stand-alone alkaline phosphatase induction comparable to that seen with 50 ng/mL rhBMP-2 in a 3 day experiment. The peak level of alkaline phosphatase induction was comparable to that achievable with BMP-2 in this assay. Data points were determined in triplicate. Statistical significance from the BMP-2 control group (*p* < 0.05) was obtained as detailed in “Materials and Methods”, and is denoted by a.

To test the ability of FK506 to initiate ligand-independent BMP/Smad signaling, C2C12 cells were treated with scaling doses of FK506 (1.5, 3, or 6 µM) or rhBMP-2, and alkaline phosphatase activity was measured after 3 days (*n* = 3). All doses of BMP-2 treatments (6, 12, and 25 ng/mL) and also all doses of FK506 treatments (1.5, 3, and 6 µM) showed significantly higher activity (*p* < 0.05) compared to NT controls. FK506 at 6 µM was capable of producing stand-alone alkaline phosphatase induction comparable to that seen with 50 ng/mL rhBMP-2 (Figure 3). The peak level of alkaline phosphatase induction by FK506 was comparable to that achievable with BMP-2 in this assay. This could be due to the potentiation of low levels of endogenous BMPs by FK506. Based on these results, we postulated that FK506 treatment alone is capable of promoting an osteogenic response from cells in a BMP-like manner. At concentrations higher than 15 µM, FK506 resulted in cell lifting and thus lower yields of cells and proteins, probably due to cellular toxicity.

### 2.3. FK506 Activation of BMP and TGF-β Pathways

In the canonical pathway, BMPs initiate the signal transduction cascade by binding to type I or type II serine/threonine kinase receptors and forming a hetero-tetrameric complex. The constitutively active type II receptor then transphosphorylates the type I receptor, and the type I receptor phosphorylates the R-Smads (Smad1/5/8) at their C-terminuses. Phosphorylated Smad1/5/8 associates with the co-Smad (Smad4), and the complex translocates to the nucleus, where it further associates with coactivators or corepressors to regulate gene expression. TGF-β also operates on a similar pathway through specific R-Smads (Smad2/3). Thus, pSmad levels correspond to the intensity of signaling.

**Figure 4:**
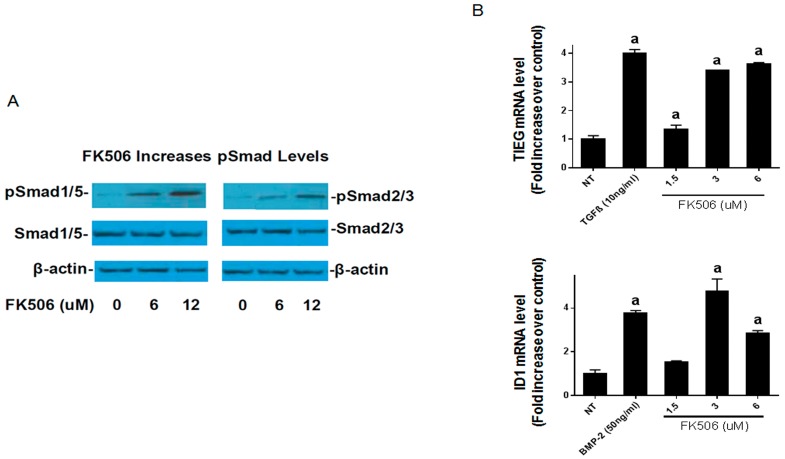
(**A**) FK506 increases BMP pSmad levels more than TGF-β pSmad levels. We treated C2C12 cells with FK506 (6 or 12 µM) and measured pSmad levels by Western blot 1 h after treatment. FK506 treatment resulted in a greater activation of the BMP receptor pSmad1/5 compared with the TGF-β receptor pSmad2 at both doses. (**B**) FK506 is capable of activating the early response genes. In order to interrogate the dose response and relative activation of BMP versus TGF-β signaling, we measured mRNA expression of early response genes in each pathway by RT-PCR. The data demonstrate induction of mRNAs for early response genes for BMP and TGF-β (*Id1* and *TIEG1*) 1 h after exposure to Ligand (BMP-2 50 ng/mL or TGF-β 10 ng/mL) or FK506 in C2C12 cells. The 3 µM dose of FK506 was capable of *Id1* or *TIEG1* induction comparable to that of 100 ng/mL of BMP-2, but higher FK506 doses may preferentially induce signals that decrease BMP pathway activation, such as TGF-β. Statistical significance for **a** denotes comparison to the no-treatment controls.

C2C12 cells were treated with FK506 (6 or 12 µM) and pSmad levels were measured by Western blot 1 h after treatment. FK506 treatment resulted in a greater activation of the BMP receptor pSmad1/5 compared with the TGF-β receptor pSmad2 at both doses (Figure 4A). These results further confirm that FK506 is capable of elevating the levels of ligand-independent phosphorylation of receptor Smads, leading to enhanced signaling.

In addition to enhancing the BMP pathway, FK506 has also been shown to bind to FKBP12 and enhance transforming growth factor β (TGF-β) type I receptor activity [30]. In order to interrogate the dose response and relative activation of BMP versus TGF-β signaling, we measured mRNA expression of *Id1* (Inhibitor of Differentiation/DNA binding, which is a member of the helix-loop-helix protein family) and *TIEG1* (TGF-β inducible early gene-1), the early response genes in the BMP and TGF-β pathways, respectively, by RT-PCR (*n* = 3). The BMP-2 (50 ng/mL) and TGF-β (10 ng/mL) treatments, and the doses of FK506 treatments (3 and 6 µM) showed significantly higher mRNA levels (*p* < 0.05) compared to NT controls. Figure 4B demonstrates that in C2C12 pluripotent cells, a treatment by FK506 dose-dependently induced mRNA levels for early-response genes for BMP and TGF-β (Id1 and TIEG1, respectively) 1 h after exposure. The 3 µM dose of FK506 was capable of Id1 induction comparable to that of 100 ng/mL of BMP-2.

### 2.4. FK506 Osteogenic Effect is Resistant to Noggin

FK506 treatment of cells results in ligand-independent activation of the BMP/Smad signaling. BMPs typically induce expression of Noggin and other extracellular inhibitors, but since FK506 effects do not require the extracellular BMP ligand, we hypothesized that FK506 would be resistant to the prototypical extracellular BMP inhibitor, Noggin.

**Figure 5:**
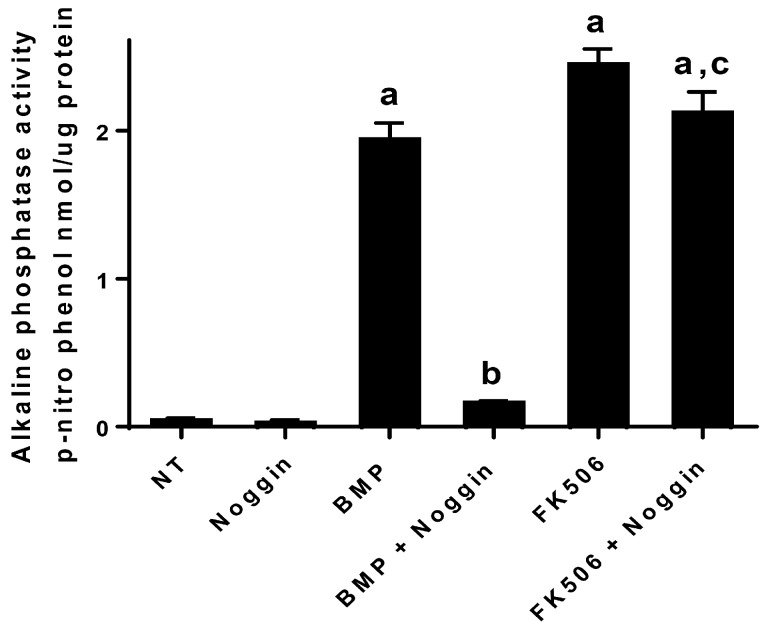
FK506 osteogenic effect is resistant to Noggin. The data demonstrate that BMP-2 (50 ng/mL) and FK506 (6 µM) increase alkaline phosphatase activity in C2C12 cells after 72 h of treatment. The BMP-induced alkaline phosphatase activity was significantly (*p* < 0.05) inhibited (~90%) by Noggin treatment (50 ng/mL) and was not significantly different from the control group (*p* < 0.05). On the other hand, the FK506 induction of alkaline phosphatase was resistant to Noggin treatment, showing a significant increase (*p* < 0.05) over no-treatment controls and no difference to the FK506 treatment alone group. Data points were determined in triplicate (*n* = 3). Statistical significances (*p* < 0.05) from the BMP-2 control, BMP and Noggin, and FK506 groups were obtained as detailed in “Materials and Methods” and are denoted by a, b and c. Statistical significance for comparison to the no-treatment control is denoted by a, b denotes comparison to the BMP alone control, and **c** denotes comparison to BMP + Noggin treatment.

Figure 5 demonstrates that BMP-2 (50 ng/mL) and FK506 (6 µM) significantly increase alkaline phosphatase activity in C2C12 cells after a 72 h treatment (*n* = 3). BMP-induced alkaline phosphatase activity was significantly (*p* < 0.05) inhibited (~90%) by Noggin treatment (50 ng/mL) and was not significantly different from the control group (*p* < 0.05). On the other hand, the FK506 induction of alkaline phosphatase was resistant to Noggin treatment, showing a significant increase (*p* < 0.05) over NT controls and no significant difference to the FK506 treatment alone group.

### 2.5. FK506 Inhibits Calcineurin Activity

**Figure 6:**
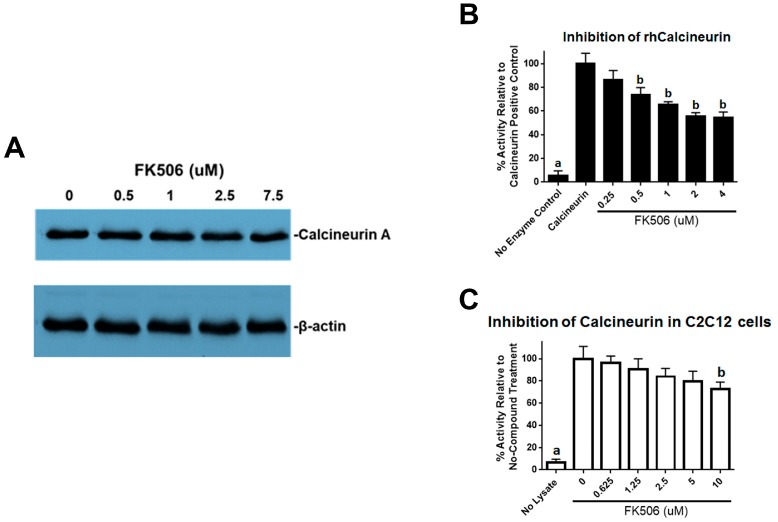
(**A**) FK506 has no effect on calcineurin A protein levels. We treated C2C12 cells with FK506 (0, 0.5, 1, 2.5, and 7.5 µM) and determined calcineurin A levels by Western blot after treatment of cells for 2 days. (**B**) FK506 treatment resulted in a dose-dependent inhibition of the phosphatase activity of a recombinant human calcineurin A in an in vitro assay described in “Materials and Methods”. Over 40% inhibition of enzyme activity was observed, even with a 2 µM concentration of FK506 (*n* = 3, *p* < 0.05) compared to the no-treatment group. Statistical significance (*n* = 3, *p* < 0.05) is denoted by **a** for comparison to the control group. Statistical significances (*n* = 3, *p* < 0.05) from the no-treatment control group to compound treatment groups are denoted by **b**. All determinations were performed in triplicate. (**C**) FK506 treatment of C2C12 cells also showed significant inhibition of calcineurin activity in cell lysates. An inhibition of over 27% was achieved with a 10 µM concentration of FK506 compared to no-treatment controls (*n* = 3, *p* < 0.05). Statistical significance (*p* < 0.05) is denoted by **a** for comparison to the control group. Statistical significances (*p* < 0.05) from the no-treatment control group to compound treatment groups are denoted by b.

Calcineurin is a Ca++–calmodulin-dependent serine/threonine protein phosphatase that was implicated to act on phosphorylated receptor Smads and thus diminish the intensity of signaling. It is a heterodimer of a ~60 kDa catalytic subunit, calcineurin A (CnA), and a 19 kDa regulatory subunit, calcineurin B (CnB) [12]. We studied the effect of FK506 on calcineurin A protein levels upon treatment of C2C12 cells with various doses of FK506 in Western blots. As shown in Figure 6A, we did not see any effect on calcineurin A protein levels with or without FK506 treatments. Then, we checked the effect of FK506 on calcineurin A phosphatase activity, both with purified recombinant human calcineurin and the C2C12 cell lysates, after various doses of FK506 treatment of cells, using the RII phosphopeptide, the best-known substrate for calcineurin. As shown in Figure 6B, the recombinant human calcineurin A (rhcalcineurin) showed significant inhibition of phosphatase activity with FK506. A dose-dependent inhibition of enzyme activity was observed with FK506. A 50% inhibition of enzyme activity was achieved with a 2 µM concentration of FK506 compared to the NT control (*p* < 0.05). Next, we assayed the C2C12 cell lysates prepared after treatment of C2C12 cells by various concentrations of FK506 for 2 days. As shown in Figure 6C, FK506 treatment of C2C12 cells showed significant inhibition (over 27%) compared to no-treatment controls (*p* < 0.05). These observations are consistent with the central hypothesis that FK506 potentiates the BMP pathway independent of BMP treatment. Inhibition of calcineurin further augments the osteogenic efficacy of FK506.

### 2.6. In Vivo Ectopic Mineralization from Local Subcutaneous Delivery of FK506

**Figure 7:**
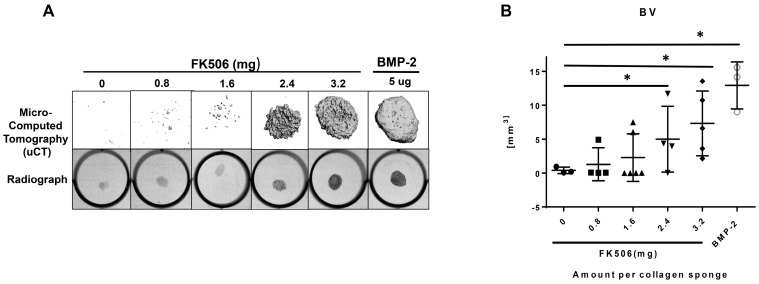
Ectopic bone induction at four weeks by FK506. There was a dose-dependent increase in bone formation. Significant differences compared with implants treated with the vehicle (DMSO)—i.e., the control group—were found at the 2.4 and 3.2 mg doses of FK506. Typical radiographs of the ectopic ossicles in the various treatment groups are also shown. The radiopaque areas of the FK506-treated groups (0.0, 0.8, 1.6, 2.4, and 3.2 mg/100 μL/disc, *n* = 3–6) were larger and more dense than those in the control group. (**A**) A digital radiographic evaluation of the collagen disc implants with varying amounts of FK506. This demonstrates the ability of FK506 to create bone with no exogenous BMP-2. (**B**) Quantitative micro-computed tomography (microCT) measurements of Bone Volume (BV) (mm^3^) that correlate with the dose of FK506 implanted (*p* < 0.05). There is a significant difference in bone volume when comparing 2.4 and 3.2 mg of FK506 as a stand-alone agent and BMP-2 to the implants treated with the vehicle (statistical significance indicated by asterisks). However, there is no significant difference between those two doses of FK506 and implants treated with BMP-2, indicating comparable bone osteoinductive capabilities.

We compared FK506 to BMP-2 delivery on a collagen sponge using a standard but challenging rat subcutaneous ectopic mineralization assay. Figure 7a demonstrates X-rays of implants excised from a rat subcutaneous pouch 4 weeks after implantation of a collagen disc with FK506 (0 to 3.2 mg in 100 μL of DMSO). FK506 delivery (2.4 and 3.2 mg, stand-alone with no BMP-2) was able to induce de novo ectopic mineralization at a level qualitatively comparable to BMP-2 (5 μg/collagen disc) delivery. Figure 7b shows quantitative micro-computed tomography (microCT) measurements of Bone Volume (BV) (mm3) with the various doses of FK506 implanted (0.0, 0.8, 1.6, 2.4, and 3.2 mg/100 μL/disc, *n* = 3–6). For FK506 treatment groups, the 1.6 mg dose was below the threshold for consistent mineralization, and had less mineral formed than in the 2.4 and 3.2 mg explants. Figure S1 shows the tissue density measurements from these specimens, and shows comparable mineral density between the samples treated with 3.2 mg FK506 and BMP-2.

**Figure 8:**
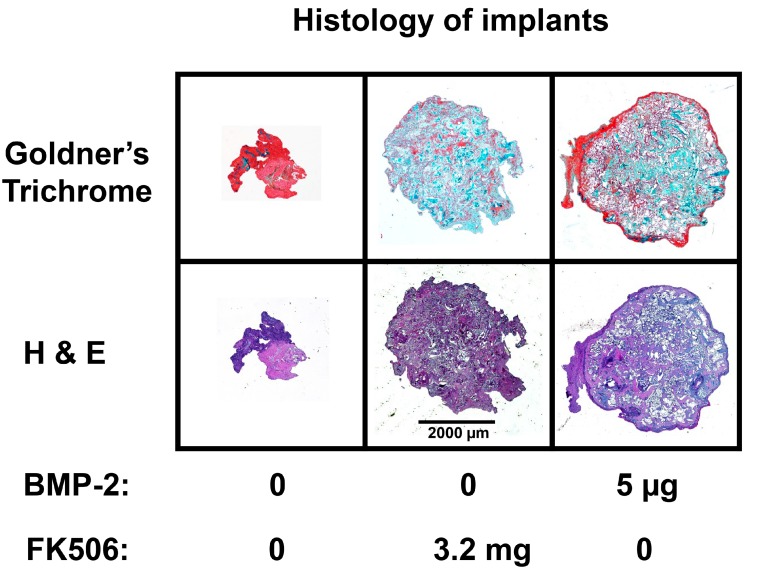
Representative images of subcutaneous collagen disk implants (4×). After four weeks, the implants were removed and embedded into paraffin. The samples were cut into 5 µm thick sections and stained with hematoxylin and eosin (H&E) and Goldner’s trichrome (green/turquoise represent bone, and red represents collagen and muscle).

A minimal dose threshold for consistent ectopic mineralization is typical of all agents utilizing the BMP signaling pathway. The FK506 doses 2.4 and 3.2 mg and BMP-2 induced significant ectopic mineralization compared to NT controls (*p* < 0.05). However, there was no significant difference between those two doses of FK506 and implants treated with BMP-2, indicating comparable mineralization capabilities. Histological analysis (Figure 8) of the tissue by H&E staining and Goldner’s trichrome staining also demonstrates ectopic mineralization of the FK506-treated samples. 3.2 mg of FK506 delivered on the collagen sponge showed comparable mineralized tissue to 5 μg of BMP-2, showing corresponding effects as with the microCT data. This in vivo experiment in rats demonstrated for the first time that FK506 induces ectopic mineralization from scratch as a stand-alone agent, even in the absence of exogenous BMP-2.

## 3. Discussion

The need for “supra-physiologic” BMP concentrations in the clinical setting stems at least in part from the fact that human MSCs are less responsive to BMP-2 at a cellular level, and the influx of MSCs to bone healing sites is slower in humans compared to rodents or primates [31,32]. As a result, higher initial loading concentrations of rhBMP-2 on the collagen carrier have been required to ensure enough BMP remains at the site when cells arrive. This larger total dose (12–40 mg in humans versus μg doses in rodents for one level spine fusion) has resulted in significant local side effects (swelling, bone resorption, nerve inflammation, etc.) that were not seen in pre-clinical studies at lower BMP concentrations [6]. These issues have resulted in an over 50% drop in usage since its peak in 2007. As such, a safe and cost-effective alternative approach to activate the BMP bone formation pathway remains of paramount clinical significance. The ligand-independent initiation of the BMP signaling pathway by FK506 has the potential to bypass these extracellular regulatory feedback mechanisms. We hypothesized that transient exposure to FK506 by local delivery in vivo would be capable of ligand-independent activation of the BMP pathway, thereby taking advantage of positive BMP feedback loops and avoiding some of the negative feedback signals.

In order to translate FK506 to a potential stand-alone local therapeutic, we started by performing a direct in vitro comparison of the dose responses of FK506 and BMP-2 to induce ALP activity in C2C12 cells. Here, we observed that an FK506 dose range from 1.5–6 µM showed a similar dose response to BMP-2 (12–50 ng·mL^−1^) for inducing ALP activity in C2C12 cells. Further, we show that FK506 is able to activate both the BMP-2 and TGF-β pathways by increasing pSmad levels and subsequently by increasing the expression of early response genes. The higher levels of pSmad reflect the increased activity of receptor kinases in both pathways, while unphosphorylated levels of the Smads remained at the same level as the controls. These data are consistent with previous studies which have shown the ability of FK506 to induce BMP signaling and osteogenic differentiation in a variety of cell types, including C17 (mouse limb bud-derived cells), C2C12 (mouse myoblasts), MC3T3 (mouse pre-osteoblasts), and mesenchymal stromal cells [19,20,21,22,23]. We further investigated the effects of FK506 on calcineurin activity and found decreased calcineurin activity in the C2C12 cells. Calcineurin has been shown to dephosphorylate pSmad1/5, and inhibition of this effect may suggest another mechanism by which FK506 could affect the BMP signaling pathway and feedback system [13].

In addition to the direct activation of BMP signaling and osteogenic differentiation by FK506, we investigated the potential feedback control of the BMP pathway by the primary extracellular inhibitor Noggin. Noggin is a natural extracellular inhibitor of BMP activity, and can be upregulated in response to BMP signaling to provide a feedback control mechanism. Noggin exerts its function by direct binding to BMP stoichiometrically. Here we show that FK506 maintained the ability to induce ALP activity in C2C12 cells even in the presence of Noggin. The lack of Noggin-induced inhibition of FK506 confirmed ligand-independent activity and bypasses the typical feedback mechanism of BMP signaling which in part necessitates the supra-physiologic dosing used for therapeutic applications.

The evidence of osteogenic activity of FK506 in vitro strongly supports its potential utility in vivo. Here we show that FK506 can be delivered in vivo on a collagen sponge to induce mineralization in an ectopic site. The quantitative microCT data showed a dose-dependent response to FK506 delivery reaching a comparable level of mineralization to that of BMP-2 delivery. This was consistent with histologic observations of the mineralized tissue. These data show that FK506 can induce ligand-independent mineralization when delivered as a stand-alone agent locally on a collagen sponge.

Previous studies have not utilized stand-alone local delivery of FK506 for bone healing applications, but instead have focused on systemic or local delivery of FK506 to enhance a different osteogenic stimulus (osteogenic cell- or BMP-2-based). Systemic administration of FK506 (1 mg/kg/d (injected) or 3 mg/kg/d (oral gavage)) was shown to enhance local MSC repair of segmental defects and systemic bone formation in the setting of inflammatory-mediated bone loss. Other studies have similarly showed that systemic delivery of FK506 could enhance the healing of bone defects treated with various combinations of osteoprogenitor cells (some genetically modified) or BMP-2 [25,26,27]. Systemic delivery of FK506 was also shown to enhance mineralization of a demineralized bone matrix carrier [28]. Separate from enhancing mineralization of implanted matrix or repairing a defect, systemic FK506 can also enhance fracture healing in impaired healing scenarios like that of multi-tissue trauma. While most bone regeneration studies have utilized systemic delivery, one study showed that local delivery of a *BMP* gene therapy and FK506 (1.6 mg/kg) could enhance the BMP-induced mineralization [29]. We found that a significantly higher dose than used previously was needed for a single local delivery to induce mineralization. The dose that produced consistent mineralization comparable to BMP-2 was 3.2 mg, which would be about 12.8 mg/kg. The lowest dose we used was 0.8 mg (about 3.2 mg/kg), which is in the range commonly used for systemic delivery (1–3 mg/kg), but this dose did not show any ectopic mineralization. The broad data in this field are beginning to provide a compelling case for the utility of FK506 in numerous bone healing applications, both as a supplement to normal or engineered healing, and, with our findings, as a direct osteoinductive stimulus.

Although there are several small molecules that are capable of enhancing bone formation, and even enhancing BMP activity, few if any, possess the osteoinductive potency of BMP itself—that is, the ability to form de novo bone in a non-bone environment. This observation for FK506, a drug that has been cleared by the FDA for systemic use as an immunosuppressant, represents an attractive target to repurpose for a new paradigm of local bone healing. In future studies, it will be critical to test this potential in pre-clinical injury and degeneration models of bone repair (segmental defect repair and spine fusion). Furthering this knowledge has the potential to enable clinically successful bone healing to be achieved, thereby making this a safe technology that could be routinely used to improve bone healing in patients.

## 4. Materials and Methods

### 4.1. Cell Culture

Mouse C2C12 cells and Dulbecco’s modified Eagle’s medium (DMEM) were purchased from ATCC (Manassas, VA, USA). The non-heat inactivated fetal bovine serum (FBS) was purchased from HyClone Laboratories, Inc. (Logan, UT, USA). The C2C12 cells at passages 4 to 7 were subcultured in T-75 cm^2^ flasks in DMEM supplemented with 10% FBS at 37 °C in 5% CO_2_ with humidification. When the flasks reached 60–70% confluence, the cells were trypsinized and seeded in triplicate at 200,000 cells/well in a 6-well plate for quantitative real-time RT-PCR and alkaline phosphatase (ALP) assays, or at 50,000 cells/well in a 12-well plate for the dual-luciferase reporter assay.

### 4.2. RNA Extraction and Reverse Transcription

The C2C12 cells were plated at a density of 200,000 cells/well in 6-well plates and grown overnight in DMEM containing 10% FBS. On day two, the culture medium was replaced with DMEM containing 2% FBS, and the cells were treated with various concentrations of the selected compound (diluted from 10 mg stock solutions prepared in DMSO) for 24 h. In control cultures, a DMSO solvent concentration of 0.01% (*v*/*v*) was applied. On day three, the medium was replaced with fresh DMEM containing 2% FBS, and the cells were treated with BMP-2 for 24 h. Total RNA was harvested using the RNeasy Mini Kit according to the manufacturer’s instructions (Qiagen, Valencia, CA, USA). The harvested RNA was digested with RNase-free DNase I (Qiagen) to remove DNA contamination. The concentration of the isolated RNA was determined by measuring the absorbance at 260 nm wave length with a spectrophotometer (Model DU 640, Beckman Coulter, Inc. Brea, CA, USA). The ratio of A260/A280 was between 1.6 and 1.8. Reverse transcription was carried out to synthesize cDNA in a 100 μL volume with 2 μg of total RNA, 10× RT buffer, 5.5 mM MgCl_2_, 2 mM deoxynucleotide triphosphate (dNTP) mixture, 0.125 μM oligo d(T), 0.125 μM random primer, 40 units of RNase inhibitor, and 125 units of MultiScribe (Applied Biosystems, Foster City, CA, USA) for 10 min at 25 °C, 30 min at 48 °C, and 5 min at 95 °C.

### 4.3. Quantitative Real-Time RT-PCR

Quantitative real-time RT-PCR was performed to determine the mRNA expression level of early marker genes of BMP and TGFβ pathways. The sequences of the primers were as follows: *Id1* (forward), 5′-GCGAGGTGGTACTTGGTCTG-3′; (reverse), 5′-GAGAGGGTGAGGCTCTGTTG-3′), *TIEG1* (forward), 5′-GCCAACCATGCTCAACTTCG-3′; (reverse), 5′-TGCAGTTTTGTTCCAGGAATACAT-3′. Twenty-five microliters of reaction volume included 5 μL of cDNA, 0.5–10 μM of each primer, and 12.5 μL of 2× SYBR green master mix (Applied Biosystems). Real-time PCR was performed with the following three-step protocol: Step 1, 50 °C for 2 min; step 2, 95 °C for 10 min; step 3, and 40 cycles of 95 °C for 15 s and 62 °C for 1 min using the 7500 real-time PCR system (Applied Biosystems). To confirm the amplification specificity, the PCR products were subjected to a dissociation curve analysis. The threshold cycles (Ct) of each reaction were normalized to those obtained for 18S mRNA using the −ΔΔ*C_t_* method (Applied Biosystems). All PCR reactions were performed in triplicate.

### 4.4. SDS-PAGE and Western Blotting

Cells were lysed to obtain total protein using Mammalian Protein Extraction Reagent (Pierce Biotechnology, Rockford, IL, USA) or lysed to obtain nuclear protein using Nuclear and Cytoplasmic Extraction Reagents (Pierce Biotechnology) according to the manufacturer’s protocol. Each sample (10 μg of protein) was mixed with NuPage loading buffer (Invitrogen, Carlsbad, CA, USA) for a total volume of 20 μL and boiled for 5 min. The proteins were separated by electrophoresis under denaturing conditions on NuPage Bis-Tris Pre-Cast gels (Invitrogen,) for 60 min at 200 Volts and transferred onto nitrocellulose membranes (Invitrogen) for 60 min at 30 Volts. After the transfer, the membranes were incubated in 25 mL of blocking buffer (5% nonfat dry milk in Tris buffered saline (TBS)) for 1 h at room temperature. After blocking, membranes were washed three times for 5 min each in 15 mL of TBS with 0.1% Tween-20 (TBST). Washed membranes were incubated with different primary antibodies in TBST overnight at 4 °C. Anti-actin antibodies were purchased from Santa Cruz Biotechnology (Santa Cruz, CA, USA). Other antibodies were purchased from Cell Signaling Technology (Beverly, MA, USA). After incubation with primary antibody, membranes were washed three times for 5 min each with 10 mL of TBST. Washed membranes were incubated with HRP-conjugated anti-rabbit or anti-mouse secondary antibodies as indicated (1:2000, Cell Signaling Technology) in 10 mL of blocking buffer with gentle agitation for 1 h at room temperature. After incubation with secondary antibodies, membranes were washed three times for 5 min each with 10 mL of TBST. Washed membranes were incubated with 5 mL of SuperSignal West Pico Western blot substrate (Pierce Biotechnology) with gentle agitation for 4 min at room temperature. Membranes were drained of excess developing solution, wrapped in plastic wrap, and exposed to X-ray films.

### 4.5. Alkaline Phosphatase (ALP) Assay

The C2C12 cells were plated at 200,000 cells/well in 6-well plates and grown overnight in DMEM containing 10% FBS. On day two, the culture medium was replaced with DMEM containing 2% FBS and the cells were treated with 0.5 uM or an indicated concentration of compound for 24 h in 2 mL culture medium. On day three, the cells were treated with a final concentration of 50 ng/mL of BMP-2 with or without compound in DMEM medium containing 2% FBS for 72 h. The cells were washed with phosphate-buffered saline (PBS) and lysed by addition of lysis buffer (10 mM Tris-HCl pH 8.0, 1 mM MgCl2, and 0.5% Triton X-100). The cell lysates were centrifuged for 5 min at 13,000× *g*. The supernatant was collected and the aliquots were assayed for ALP activity and protein amount. The ALP activity was measured in triplicate using an ALP assay kit (Sigma-Aldrich) in microtiter plates. The protein amount was determined with Bio-Rad protein assay reagent (Bio-Rad, Hercules, CA, USA) using bovine serum albumin (BSA) as a standard. The ALP activity (nmoles of *p*-nitrophenol per mL) was normalized to the protein amount (nmoles of *p*-nitrophenol per μg).

### 4.6. Calcineurin Activity Assay

Calcineurin activity was measured using a calcineurin cellular activity assay (Enzo Life Sciences, New York, NY, USA) according to manufacturer’s protocols. Mouse myoblastic C2C12 cell lysates were placed in a desalting column to remove excess phosphates and nucleotides. Total phosphatase activity was determined in the samples by incubating with a calcineurin-specific substrate (RII phosphopeptide, with sequence Asp-Leu-Asp-Val-Pro-Ile-Pro-Gly-Arg-Phe-Asp-Arg-Arg-Val-pSer-Val-Ala-Ala-Glu, designed from a subunit of the bovine cAMP-dependent protein kinase). A purified recombinant human calcineurin was used as a positive control. The detection of free-phosphate released was accomplished with Biomol Green reagent by monitoring absorbance at 620 nm in a SpectraMax M2 microplate reader (Molecular Devices, San Jose, CA, USA).

### 4.7. Ectopic Bone Formation Model

All animal procedures were approved by the local Institutional Animal Care and Use Committee. The FK506 compound was first tested in a standard athymic rat chest ectopic bone formation model using of a 5 μg/disc dose of rhBMP-2 as a positive control to induce bone formation consistently. rhBMP-2 or FK506 were loaded with use of a pipette onto sterile bovine Type-I collagen disks (8 mm in diameter and 3 mm thick; Kensey Nash, Exton, PA, USA) in a biosafety cabinet. The disks were then transported in a sterile container to the surgical operating room. Each implant was loaded with a total volume of 100 uL solution containing 5 μg of rhBMP-2 (Medtronic, Minneapolis, Minnesota) or 100 μL of stock concentrations of 0, 10, 20, 30, and 40 mM of FK506 (corresponding to 0.8, 1.6, 2.4, and 3.2 mg of dry weight, respectively) solubilized in the organic solvent dimethyl sulfoxide (DMSO, Sigma-Aldrich) (*n* = 4 for each). In a pilot experiment, 10% to 100% DMSO was determined to have no effect on rhBMP-2-induced ectopic bone formation (data not shown).

Male athymic nude five to six-week-old rats (Harlan Laboratories, Indianapolis, IN, USA) were anesthetized with 1% to 2% isoflurane mixed with oxygen at a flow rate of 0.5 to 1 L/min and maintained during surgery with the same dose. Surgery was performed with the animal positioned supine on a circulating-water heating pad. Four 1 cm transverse incisions were made about 3 cm apart on the chest of each rat, and subcutaneous pockets were created by blunt dissection with scissors. The implants were inserted into the pockets, and closure was accomplished with closely spaced interrupted absorbable polyglactin-910 sutures (Vicryl; Ethicon, Johnson & Johnson, Somerville, NJ, USA).

The rats were housed in autoclaved cages (two per cage) that had a microisolator top and contained autoclaved bedding, and they were given autoclaved food and water ad libitum. Cages were changed in a biosafety cabinet. All of the rats fed well after the surgery. There were no postoperative complications associated with the surgical procedure. The rats were killed four weeks postoperatively. The implants were harvested and were evaluated with manual palpation, high-resolution digital radiography, and non-decalcified histological analysis.

### 4.8. Digital Radiographic Evaluation

X-ray scanning (In-Vivo Xtreme, Bruker Corp., Billerica, MA, USA) was performed on the subcutaneous implants after fixation. The scans were executed with an exposure time of 1.2 s and a voltage of 45 kV. To assess bone formation, micro-computed tomography (microCT) scans (Micro-CT40, Scanco Medical, Bruttisellen, Switzerland) were performed. Samples were scanned with a 30 µM voxel size at a voltage of 45 kVp and a current of 177 μA. Three-dimensional reconstructions were obtained from evaluations of 500 slices of two-dimensional X-ray images.

### 4.9. Histological Analysis of Subcutaneous Ectopic Bone Formation

After euthanasia, at 4 weeks post-surgery, the subcutaneous implants were fixed with 10% formalin. Following fixation, the implants were washed and placed into a processor which dehydrated the samples sequentially in 70%, 95% and 100% alcohol, followed by xylene. The samples were then embedded in paraffin and cut into slices of 5 microns using a microtome (Accu-Cut SRM 200 Rotary Microtomoe, Sakura Finetek USA, Torrance, CA, USA). Slides were stained with Hematoxylin and Eosin (H&E) and Goldner’s trichrome (Sigma-Aldrich). Images were obtained with Lionheart LX (Biotek Instruments Inc., Winooski, VT, USA) at 4× and captured using Gen 5 software (Biotek Instruments Inc., Winooski, VT, USA)).

### 4.10. Statistics and Calculations

Results are presented as the mean of three determinations (*n*) with error bars representing the standard error of the mean (SEM). Experimental results that are visually represented are from consistent experiments where one representative experimental result is shown. Statistical significance (*p* < 0.05) was calculated using a one-way analysis of variance (ANOVA) with Bonferroni Post Hoc test (equal variances assumed) or Dunnett’s T3 Post Hoc test (equal variances not assumed) using Statistical Products for Social Sciences Version 16.0 (SPSS 16.0, IBM, Quarry Bay, HK, China) for Windows to compare various treatments in a multi-group analysis. A statistical probability of *p* < 0.05 was considered significant and is denoted as (a,b,c) in the figures.

## Supplementary Materials

Supplementary materials can be found at https://www.mdpi.com/1422-0067/20/8/1900/s1.

## Abbreviations

BMP	Bone Morphogenetic Protein
MSC	Mesenchymal Stem Cell
rhBMP	Recombinant Human Bone Morphogenetic Protein
BMPR	Bone Morphogenetic Protein Receptor
FKBP	FK-Binding-Protein
NT	No Treatment

## References

[1] DePalma A.F., Rothman R.H. (1992). The nature of pseudoarthrosis. 1968. Clin. Orthop. Relat. Res..

[2] Cheng H., Jiang W., Phillips F.M., Haydon R.C., Peng Y., Zhou L., Luu H.H., An N., Breyer B., Vanichakarn P. (2003). Osteogenic activity of the fourteen types of human bone morphogenetic proteins (BMPs). J. Bone Joint Surg. Am..

[3] Ackerman S.J., Mafilios M.S., Polly D.W. (2002). Economic evaluation of bone morphogenetic protein versus autogenous iliac crest bone graft in single-level anterior lumbar fusion: An evidence-based modeling approach. Spine (Phila Pa 1976).

[4] Boden S.D., Zdeblick T.A., Sandhu H.S., Heim S.E. (2000). The use of rhBMP-2 in interbody fusion cages. Definitive evidence of osteoinduction in humans: A preliminary report. Spine (Phila Pa 1976).

[5] Fujimura K., Bessho K., Okubo Y., Kusumoto K., Segami N., Iizuka T. (2002). The effect of fibroblast growth factor-2 on the osteoinductive activity of recombinant human bone morphogenetic protein-2 in rat muscle. Arch. Oral Biol..

[6] Lee K.B., Taghavi C.E., Murray S.S., Song K.J., Keorochana G., Wang J.C. (2012). BMP induced inflammation: A comparison of rhBMP-7 and rhBMP-2. J. Orthop. Res..

[7] Crawford C.H., Carreon L.Y., McGinnis M.D., Campbell M.J., Glassman S.D. (2009). Perioperative complications of recombinant human bone morphogenetic protein-2 on an absorbable collagen sponge versus iliac crest bone graft for posterior cervical arthrodesis. Spine (Phila Pa 1976).

[8] Smucker J.D., Rhee J.M., Singh K., Yoon S.T., Heller J.G. (2006). Increased swelling complications associated with off-label usage of rhBMP-2 in the anterior cervical spine. Spine (Phila Pa 1976).

[9] Singhatanadgit W., Salih V., Olsen I. (2006). Up-regulation of bone morphogenetic protein receptor IB by growth factors enhances BMP-2-induced human bone cell functions. J. Cell Physiol..

[10] Hassel S., Schmitt S., Hartung A., Roth M., Nohe A., Petersen N., Ehrlich M., Henis Y.I., Sebald W., Knaus P. (2003). Initiation of Smad-dependent and Smad-independent signaling via distinct BMP-receptor complexes. J. Bone Joint Surg. Am..

[11] Inman G.J., Nicolas F.J., Callahan J.F., Harling J.D., Gaster L.M., Reith A.D., Laping N.J., Hill C.S. (2002). SB-431542 is a potent and specific inhibitor of transforming growth factor-beta superfamily type I activin receptor-like kinase (ALK) receptors ALK4, ALK5, and ALK7. Mol. Pharmacol..

[12] Stewart A.A., Ingebritsen T.S., Manalan A., Klee C.B., Cohen P. (1982). Discovery of a Ca^2+^-and calmodulin-dependent protein phosphatase: Probable identity with calcineurin (CaM-BP80). FEBS Lett..

[13] Cho A., Tang Y., Davila J., Deng S., Chen L., Miller E., Wernig M., Graef I.A. (2014). Calcineurin signaling regulates neural induction through antagonizing the BMP pathway. Neuron.

[14] Wang T., Donahoe P.K. (2004). The immunophilin FKBP12: A molecular guardian of the TGF-beta family type I receptors. Front. Biosci..

[15] Wong E., Sangadala S., Boden S.D., Yoshioka K., Hutton W.C., Oliver C., Titus L. (2013). A novel low-molecular-weight compound enhances ectopic bone formation and fracture repair. J. Bone Joint Surg. Am..

[16] Venkataramanan R., Swaminathan A., Prasad T., Jain A., Zuckerman S., Warty V., McMichael J., Lever J., Burckart G., Starzl T. (1995). Clinical pharmacokinetics of tacrolimus. Clin. Pharmacokinet.

[17] Kershner R.P., Fitzsimmons W.E. (1996). Relationship of FK506 whole blood concentrations and efficacy and toxicity after liver and kidney transplantation. Transplantation.

[18] Kang C.B., Hong Y., Dhe-Paganon S., Yoon H.S. (2008). FKBP family proteins: Immunophilins with versatile biological functions. Neurosignals.

[19] Byun Y.K., Kim K.H., Kim S.H., Kim Y.S., Koo K.T., Kim T.I., Seol Y.J., Ku Y., Rhyu I.C., Lee Y.M. (2012). Effects of immunosuppressants, FK506 and cyclosporin A, on the osteogenic differentiation of rat mesenchymal stem cells. J. Periodontal Implant. Sci.

[20] Tang L., Ebara S., Kawasaki S., Wakabayashi S., Nikaido T., Takaoka K. (2002). FK506 enhanced osteoblastic differentiation in mesenchymal cells. Cell Biol. Int..

[21] Kugimiya F., Yano F., Ohba S., Igawa K., Nakamura K., Kawaguchi H., Chung U.I. (2005). Mechanism of osteogenic induction by FK506 via BMP/Smad pathways. Biochem. Biophys. Res. Commun..

[22] Nakamura T., Shinohara Y., Momozaki S., Yoshimoto T., Noguchi K. (2013). Co-stimulation with bone morphogenetic protein-9 and FK506 induces remarkable osteoblastic differentiation in rat dedifferentiated fat cells. Biochem. Biophys. Res. Commun..

[23] Darcy A., Meltzer M., Miller J., Lee S., Chappell S., Ver Donck K., Montano M. (2012). A novel library screen identifies immunosuppressors that promote osteoblast differentiation. Bone.

[24] Spiekerkoetter E., Tian X., Cai J., Hopper R.K., Sudheendra D., Li C.G., El-Bizri N., Sawada H., Haghighat R., Chan R. (2013). FK506 activates BMPR2, rescues endothelial dysfunction, and reverses pulmonary hypertension. J. Clin. Investig..

[25] Voggenreiter G., Assenmacher S., Kreuzfelder E., Wolf M., Kim M.R., Nast-Kolb D., Schade F.U. (2000). Immunosuppression with FK506 increases bone induction in demineralized isogeneic and xenogeneic bone matrix in the rat. J. Bone Miner. Res..

[26] De La Vega R.E., De Padilla C.L., Trujillo M., Quirk N., Porter R.M., Evans C.H., Ferreira E. (2018). Contribution of Implanted, Genetically Modified Muscle Progenitor Cells Expressing BMP-2 to New Bone Formation in a Rat Osseous Defect. Mol. Ther..

[27] Liu F., Ferreira E., Porter R.M., Glatt V., Schinhan M., Shen Z., Randolph M.A., Kirker-Head C.A., Wehling C., Vrahas M.S. (2015). Rapid and reliable healing of critical size bone defects with genetically modified sheep muscle. Eur. Cell Mater..

[28] Hurtgen B.J., Henderson B.E.P., Ward C.L., Goldman S.M., Garg K., McKinley T.O., Greising S.M., Wenke J.C., Corona B.T. (2017). Impairment of early fracture healing by skeletal muscle trauma is restored by FK506. BMC Musculoskelet Disord..

[29] Kaihara S., Bessho K., Okubo Y., Sonobe J., Kawai M., Iizuka T. (2004). Simple and effective osteoinductive gene therapy by local injection of a bone morphogenetic protein-2-expressing recombinant adenoviral vector and FK506 mixture in rats. Gene Ther..

[30] Aghdasi B., Ye K., Resnick A., Huang A., Ha H.C., Guo X., Dawson T.M., Dawson V.L., Snyder S.H. (2011). FKBP12, the 12-kDa FK506-binding protein, is a physiologic regulator of the cell cycle. Proc. Natl. Acad. Sci. USA.

[31] Boden S.D., Martin G.J., Morone M.A., Ugbo J.L., Moskovitz P.A. (1999). Posterolateral lumbar intertransverse process spine arthrodesis with recombinant human bone morphogenetic protein 2/hydroxyapatite-tricalcium phosphate after laminectomy in the nonhuman primate. Spine (Phila Pa 1976).

[32] Osyczka A.M., Diefenderfer D.L., Bhargave G., Leboy P.S. (2004). Different effects of BMP-2 on marrow stromal cells from human and rat bone. Cells Tissues Organs.

