# Correction: Sangadala, S. et al. FK506 Induces Ligand-Independent Activation of the Bone Morphogenetic Protein Pathway and Osteogenesis. *Int. J. Mol. Sci.* 2019, *20*, 1900

**DOI:** 10.3390/ijms21176287

**Published:** 2020-08-31

**Authors:** Sreedhara Sangadala, Emily J. Devereaux, Steven M. Presciutti, Scott D. Boden, Nick J. Willett

**Affiliations:** 1 Atlanta VA Medical Center, Decatur, GA 30033, USA; steven.presciutti@emory.edu; 2 Department of Orthopaedics, Emory University School of Medicine, Atlanta, GA 30329, USA; emily.foerster@emory.edu (E.J.D.); sboden@emory.edu (S.D.B.)

The authors wish to make the following corrections to this paper [1]:

The author name “Nick J. Willet” should be “Nick J. Willett”.

We have recently become aware of errors in Section 4.7 of the published paper regarding our ectopic bone formation model. We incorrectly stated that the in vivo subcutaneous implantation model utilized an immunocompromised athymic strain of rats from Harlan Labs; the strain used in the study was Sprague Dawley rats from Charles River Laboratories. Section 4.7 should read as follows.

All animal procedures were approved by the local Institutional Animal Care and Use Committee. The FK506 compound was first tested in a standard rat chest ectopic bone formation model using a 5 μg/disc dose of rhBMP-2 as a positive control to induce bone formation consistently. rhBMP-2 or FK506 were loaded with the use of a pipette onto sterile bovine Type-I collagen disks (8 mm in diameter and 3 mm thick; Kensey Nash, Exton, PA, USA) in a biosafety cabinet. The disks were then transported in a sterile container to the surgical operating room. Each implant was loaded with a total volume of 100 uL solution containing 5 μg of rhBMP-2 (Medtronic, Minneapolis, MN, USA) or 100 μL of stock concentrations of 0, 10, 20, 30, and 40 mM of FK506 (corresponding to 0.8, 1.6, 2.4, and 3.2 mg of dry weight, respectively) solubilized in the organic solvent dimethyl sulfoxide (DMSO, Sigma-Aldrich) (*n* = 4 for each). In a pilot experiment, 10% to 100% DMSO was determined to have no effect on rhBMP-2-induced ectopic bone formation (data not shown).

Male Sprague Dawley five to six-week-old rats (Charles River Laboratories, Wilmington, MA, USA) were anesthetized with 3% to 5% isoflurane mixed with oxygen at a flow rate of 0.5 to 1 L/min and maintained during surgery with a dose of 1% to 2%. Surgery was performed with the animal positioned supine on a circulating-water heating pad. Two to four 1 cm transverse incisions were made about 3 cm apart on the chest of each rat, and subcutaneous pockets were created by blunt dissection with scissors. The implants were inserted into the pockets, and closure was accomplished with either 3-0 or 4-0 resorbable sutures (Vicryl; Ethicon, Johnson & Johnson, Somerville, NJ, USA) and/or skin clips. The rats were euthanized four weeks postoperatively. The implants were harvested and evaluated with manual palpation, high-resolution digital radiography, and non-decalcified histological analysis.

These corrections do not change the overall outcomes of the study. The incorrect strain that is listed is an immunocompromised animal, whereas we used an immunocompetent strain. This is an important correction given that the drug, FK506, utilized in this study can have an effect on immune cells. The authors would like to apologize for any inconvenience caused to the readers by these changes.
