# Supplementary material for: FK506 Induces Ligand-Independent Activation of the Bone Morphogenetic Protein Pathway and Osteogenesis
Source: Int J Mol Sci. 2019 Apr 17;20(8):1900. doi: 10.3390/ijms20081900 (PMC6515024; doi:10.3390/ijms20081900)
Supplement: Supplementary file 1 [file ijms-20-01900-s001.pdf]

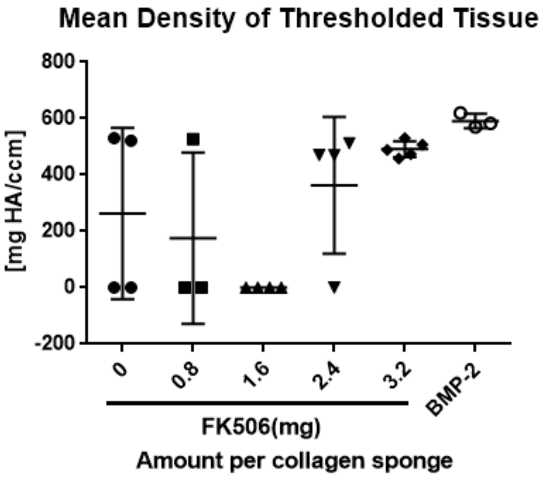

Supplementary Fig. 1- Mean density of thresholded tissue above 125 measured in mg of hydroxyapatite per cubic centimeter. This corresponds to the bone volumes found in figure 7. There were very small volumes of tissue from the control group and the 0.8 mg of FK506 group resulted in tissue above the threshold. The density of the newly formed tissue in the 3.2 mg treatment group is comparable to that of the density of the BMP-2 treated samples.
